# Unveiling the microbiome during post-partum uterine infection: a deep shotgun sequencing approach to characterize the dairy cow uterine microbiome

**DOI:** 10.1186/s42523-023-00281-5

**Published:** 2023-11-20

**Authors:** Carl Basbas, Adriana Garzon, Cory Schlesener, Machteld van Heule, Rodrigo Profeta, Bart C. Weimer, Noelia Silva-del-Rio, Barbara A. Byrne, Betsy Karle, Sharif S. Aly, Fabio S. Lima, Richard V. Pereira

**Affiliations:** 1 Department of Population Health & Reproduction, School of Veterinary Medicine, University of California, Davis, CA USA; 2 Department of Pathology, Microbiology & Immunology, School of Veterinary Medicine, University of California, Davis, USA; 3 Cooperative Extension, Division of Agriculture and Natural Resources, University of California, Orland, CA USA; 4 Veterinary Medicine Teaching and Research Center, School of Veterinary Medicine, University of California, Davis, Tulare, CA USA; 5 Department of Morphology, Imaging, Orthopedics, Rehabilitation and Nutrition, Faculty of Veterinary Medicine, University of Ghent, Merelbeke, Belgium; 6 100K Pathogen Genome Project, University of California, Davis, CA USA

**Keywords:** Uterine microbiome, Microbiota, Metritis, Diversity

## Abstract

**Background:**

The goal of this study was to assess the microbial ecology and diversity present in the uterus of post-partum dairy cows with and without metritis from 24 commercial California dairy farms using shotgun metagenomics. A set subset of 95 intrauterine swab samples, taken from a larger selection of 307 individual cow samples previously collected, were examined for α and β diversity and differential abundance associated with metritis. Cows within 21 days post-partum were categorized into one of three clinical groups during sample collection: control (CT, n = 32), defined as cows with either no vaginal discharge or a clear, non-purulent mucus vaginal discharge; metritis (MET, n = 33), defined as a cow with watery, red or brown colored, and fetid vaginal discharge; and purulent discharge cows (PUS, n = 31), defined as a non-fetid purulent or mucopurulent vaginal discharge.

**Results:**

All three clinical groups (CT, MET, and PUS) were highly diverse, with the top 12 most abundant genera accounting for 10.3%, 8.8%, and 10.1% of mean relative abundance, respectively. The α diversity indices revealed a lower diversity from samples collected from MET and PUS when compared to CT cows. PERMANOVA statistical testing revealed a significant difference (*P* adjusted < 0.01) in the diversity of genera between CT and MET samples (R2 = 0.112, *P* = 0.003) and a non-significant difference between MET and PUS samples (R2 = 0.036, *P* = 0.046). ANCOM-BC analysis revealed that from the top 12 most abundant genera, seven genera were increased in the natural log fold change (LFC) of abundance in MET when compared to CT samples: *Bacteroides, Clostridium, Fusobacterium, Phocaeicola, Porphyromonas*, *Prevotella,* and *Streptococcus*. Two genera, *Dietzia* and *Microbacterium*, were decreased in natural LFC of abundance when comparing MET (regardless of treatment) and CT, while no changes in natural LFC of abundance were observed for *Escherichia, Histophilus,* and *Trueperella*.

**Conclusions:**

The results presented here, are the current deepest shotgun metagenomic analyses conducted on the bovine uterine microbiome to date (mean of 256,425 genus-level reads per sample). Our findings support that uterine samples from cows without metritis (CT) had increased α-diversity but decreased β-diversity when compared to metritis or PUS cows, characteristic of dysbiosis. In summary, our findings highlight that MET cows have an increased abundance of *Bacteroides*, *Porphyromonas*, and *Fusobacterium* when compared to CT and PUS, and support the need for further studies to better understand their potential causal role in metritis pathogenesis.

**Supplementary Information:**

The online version contains supplementary material available at 10.1186/s42523-023-00281-5.

## Introduction

As the fourth most common health issue in cows as identified by U.S. producers, metritis remains a major detriment to the American dairy industry [1]. Metritis is a uterine disease in cattle and typically occurs within 21 days post-partum, characterized by an enlarged uterus, fever, and fetid, watery red-brown uterine discharge [2]. Metritis negatively impacts milk production, reproductive performance, and increases the risk of culling [3]. The economic impacts of these production issues cost producers a mean of $511 per case of metritis [4]. In North America, metritis is estimated to affect 10 to 30% of post-partum dairy cows [5, 6].

Generally, bacteria are frequently implicated as the cause of bovine metritis. Traditional, culture-based methods have often isolated certain bacteria from uterine swabs collected from cows with metritis, in particular, *Escherichia coli*, *Trueperella pyogenes*, *Fusobacterium necrophorum*, and *Prevotella melaninogenica* [7, 8]. However, such studies were limited to only those microbes that could be isolated and identified while growing on the media type and in atmospheric conditions provided. With the advent of culture-independent 16S rRNA gene sequencing, a substantially larger array of microbes was identified, and additional bacteria were associated with metritis, including those belonging to the genera *Bacteroides* and *Porphyromonas* [9–11]. The 16S rRNA-based studies also identified bacteria potentially associated with uterine health, albeit with occasionally conflicting findings. For example, the species belonging to the genus *Escherichia* are well-known uterine pathogens as evidenced by culture-based and animal-infection studies [12]. Yet, various studies have also demonstrated either an association between *E. coli* and uterine health or found little to no reads matching *E. coli* from uterine samples taken from cows with metritis [9, 13].

Despite the inability of 16S rRNA-based approaches to reach a uniform agreement on a specific etiology for metritis, these analyses have allowed for the study of the overall community dynamics within the uterine microbiome in addition to increases or decreases of relative abundance for specific taxa. Common ecology metrics from microbial community analysis studies often estimate α-diversity (diversity of microbes within a sample) and β-diversity (diversity of microbes between samples) [14]. Again, 16S rRNA-based studies are inconclusive on whether uterine samples taken from cows with metritis have significant changes in α-diversity. Some studies reported decreased α-diversity in samples taken from cows with metritis compared to healthy cows [9, 10, 15]; while other studies reported no statistical difference in α-diversity metrics between samples collected from with metritis and healthy cows [11, 16, 17]. One possible explanation for the disagreement between these studies is the wide range of sample size and sequencing depth, with one of the larger studies [9] (n = 60) resulting in nearly 5 million 16S reads for all samples, while one of the smaller studies [17 (n = ]28) resulted in approximately 27,000 16S reads. As metrics for the estimation of α-diversity are heavily biased when taxa are unobserved, the ability of shotgun metagenomics to identify low abundance taxa more accurately and without the biases introduced by the necessary PCR amplification used in 16S rRNA methods may prove useful in discerning the community interactions within the uterine microbiome [18, 19].

The β-diversity of uterine microbiota from cows with and without metritis is also frequently measured in uterine microbiome studies. Typically, various comparative metrics (e.g. Bray–Curtis or UniFrac) analyzing the similarity or dissimilarity of the microbiota occurrence are calculated; ordination plots based on these metrics (e.g. Principal Coordinate Analysis and Non-metric multidimensional scaling) display the microbiota diversity and are generally paired with statistical analyses (e.g. PERMANOVA and ANOSIM) to determine significance [14]. While the 16S rRNA-based studies examined previously disagreed on the α-diversity of healthy uterine microbiota and from uterus affected with metritis, those that did analyze β-diversity all concluded that the uterine microbiome from healthy and metric cows was significantly different [10, 15–17].

We previously evaluated intrauterine *E. coli* isolated from 307 dairy cows with and without metritis throughout California for phenotypic antimicrobial resistance (AMR) and analyzed risk factors impacting the isolation of intrauterine *E. coli* [20]. The objective of this study was to use shotgun metagenomic analyses on a subset of intrauterine swabs (n = 95) to assess the microbial ecology and diversity of microbes observed in the uterus during metritis and when the uterus is not affected by metritis. We hypothesized that cows with metritis would have a distinct microbial composition, characterized by a microbiome dysbiosis when compared to cows without metritis or categorized as PUS.

## Results

### Intrauterine microbiome diversity analysis

**Fig. 1:**
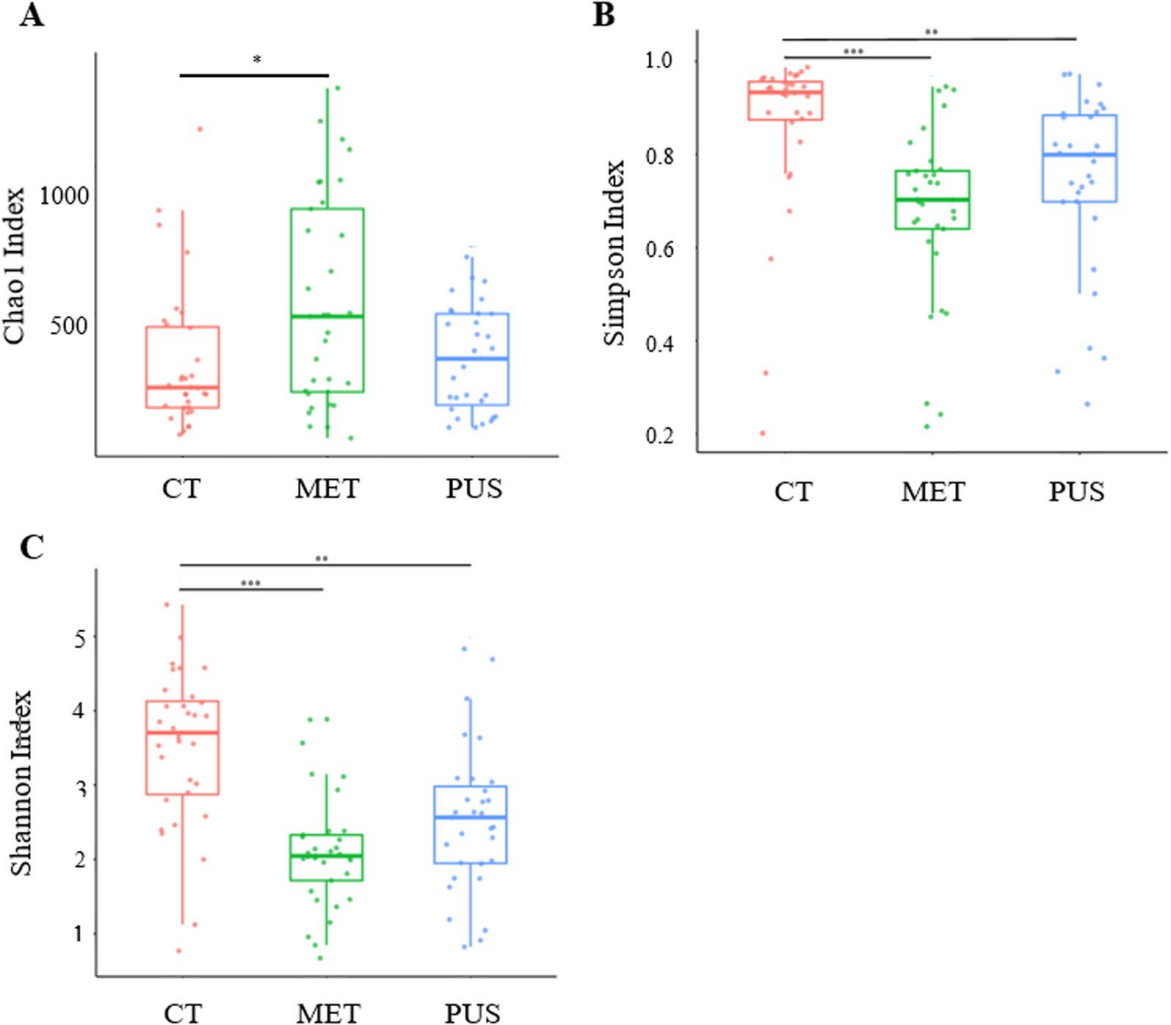
Bovine intrauterine swab microbiome α diversity. Genus-level distributions for **A** Chao1, **B** Simpson, and **C** Shannon indices, comparing clinical groups (CT = 32, MET = 33, and PUS = 31). For microbiome α diversity data, only Shannon data were normally distributed (Shapiro–Wilk *p* value = 0.073). Horizontal black lines indicate significant pairwise comparisons of clinical groups had significantly different mean Chao1 and Simpson values based on Wilcoxon Sum Rank Test or significantly different mean Shannon values based on Tukey–Kramer HSD *p* < 0.05 (*), *p* < 0.001 (**), and *p* < 0.0001 (***); *p* < 0.05 was considered a significant difference

The α diversity was assessed by genera using Chao1, Simpson, and the Shannon index (Fig. 1). The Chao1 index estimates were significantly increased for MET compared to CT samples (*p* = 0.021) (Fig. 1A). Simpson index values were significantly decreased between CT and PUS samples (*p* = 0.0005) and CT and MET samples (*p* < 0.0001) (Fig. 1B). Shannon index values were significantly different between CT and PUS samples (*p* = 0.0003) and CT and MET samples (*p* < 0.0001) (Fig. 1C).

**Fig. 2:**
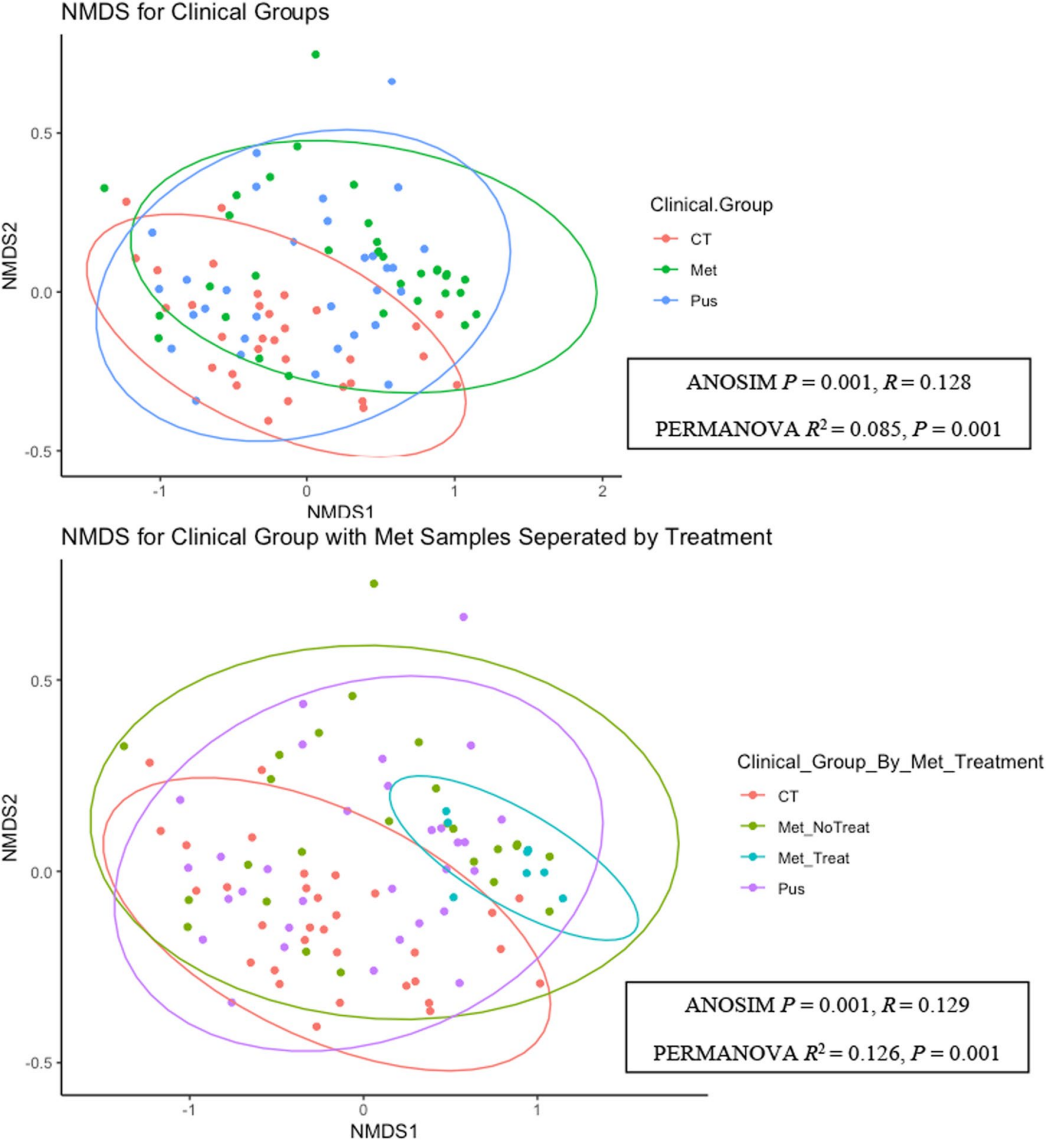
Nonmetric multidimensional scaling (NMDS) based on Bray–Curtis (BC) dissimilarity of cumulative sum scaling normalized genus-level read counts of bovine intrauterine microbiome. Ellipses correspond to 95% confidence interval. **A** NMDS ordination by three clinical groups of cows sampled (CT = 32, MET = 33, and PUS = 31) (ANOSIM- analysis of similarities, *p* = 0.001, *R* = 0.128; PERMANOVA- permutational multivariate analysis of variance, *R*^2^ = 0.085, *p* = 0.001) **B** NMDS ordination by clinical groups of cows samples with MET cows (i.e., cows with clinical signs of metritis) stratified by treatment with antimicrobials or not (CT = 32, MET_No Treatment = 23, MET_Treatment = 10, and PUS = 31) (ANOSIM *p* = 0.001, *R* = 0.129; PERMANOVA *R*^2^ = 0.126, *p* = 0.001)

Bray–Curtis dissimilarity distances calculated from CSS normalized read count data were used to create NMDS ordinations to visualize β diversity. Two NMDS ordinations were created to identify differences between clinical groups of cows sampled (Fig. 2A) and between the same clinical groups, but with samples from MET cows separated by whether cows sampled received antimicrobial treatment (Fig. 2B). Three-dimensional scatterplots of both NMDS ordinations were also created to help better visualize significant differences between clinical groups (Additional file 1: Fig. 3A) and clinical groups with MET separated by antimicrobial treatment (Additional file 1: Fig. 3B). PERMANOVA statistical testing revealed a significant difference (*P* adjusted < 0.01) in the diversity of genera between CT and MET samples (*R*^2^ = 0.112, *P* = 0.003) and a non-significant difference between MET and PUS samples (*R*^2^ = 0.036, *P* = 0.046). When analyzing NMDS with MET separated by antimicrobial treatment, pairwise PERMANOVA revealed significant differences between CT v. MET_NoTreat (*R*^2^ = 0.075, *P* = 0.006), CT v. MET_Treat (*R*^2^ = 0.242, *P* = 0.003), and PUS v. MET_Treat (*R*^2^ = 0.155, *P* = 0.003) (Additional file 2: Table 1). In comparison, pairwise ANOSIM revealed significant differences between only CT v. MET_NoTreat (*P* = 0.003) and CT v. MET_Treat (*P* = 0.003) (Additional file 2: Table 2). There were 1301 total genera detected by Kraken2/Bracken analysis (at least 100 sequence reads per million, in at least one sample). The total apparent taxa abundances are visualized in a heatmap present in Additional file 1: Fig. 3.

### Differentially abundant taxa

At the genus level, all three clinical groups (CT, MET, and PUS) were highly diverse with the top 12 most abundant genera only accounting for 10.3%, 8.8%, and 10.1% of mean relative abundance, respectively. In other words, all other genera made up 89.7%, 91.2%, and 89.9% of CT, MET, and PUS sample microbiota. Within the top 12 most abundant genera, *Bacteroides* was the most abundant (1.2% of CT, 1% of MET, 1.1% of PUS), while the least abundant was *Trueperella* (0.64% of CT, 0.56% of MET, 0.64% of PUS).

### Comparative abundance across treatment groups

**Fig. 3:**
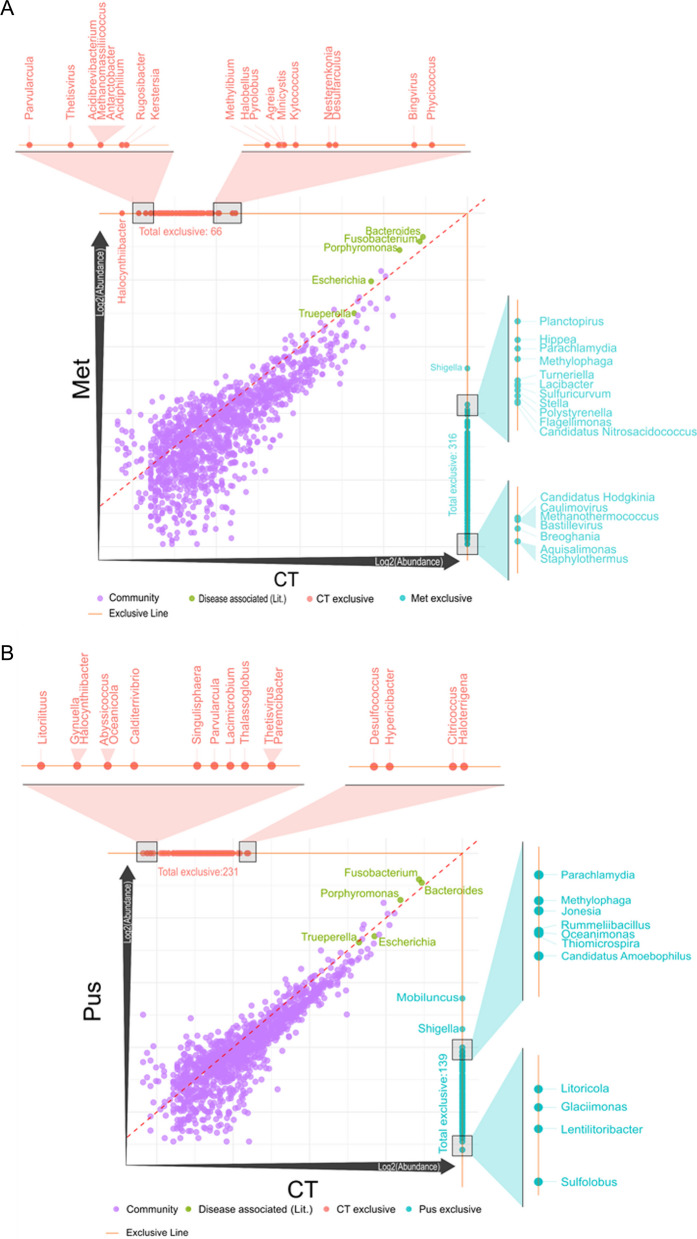

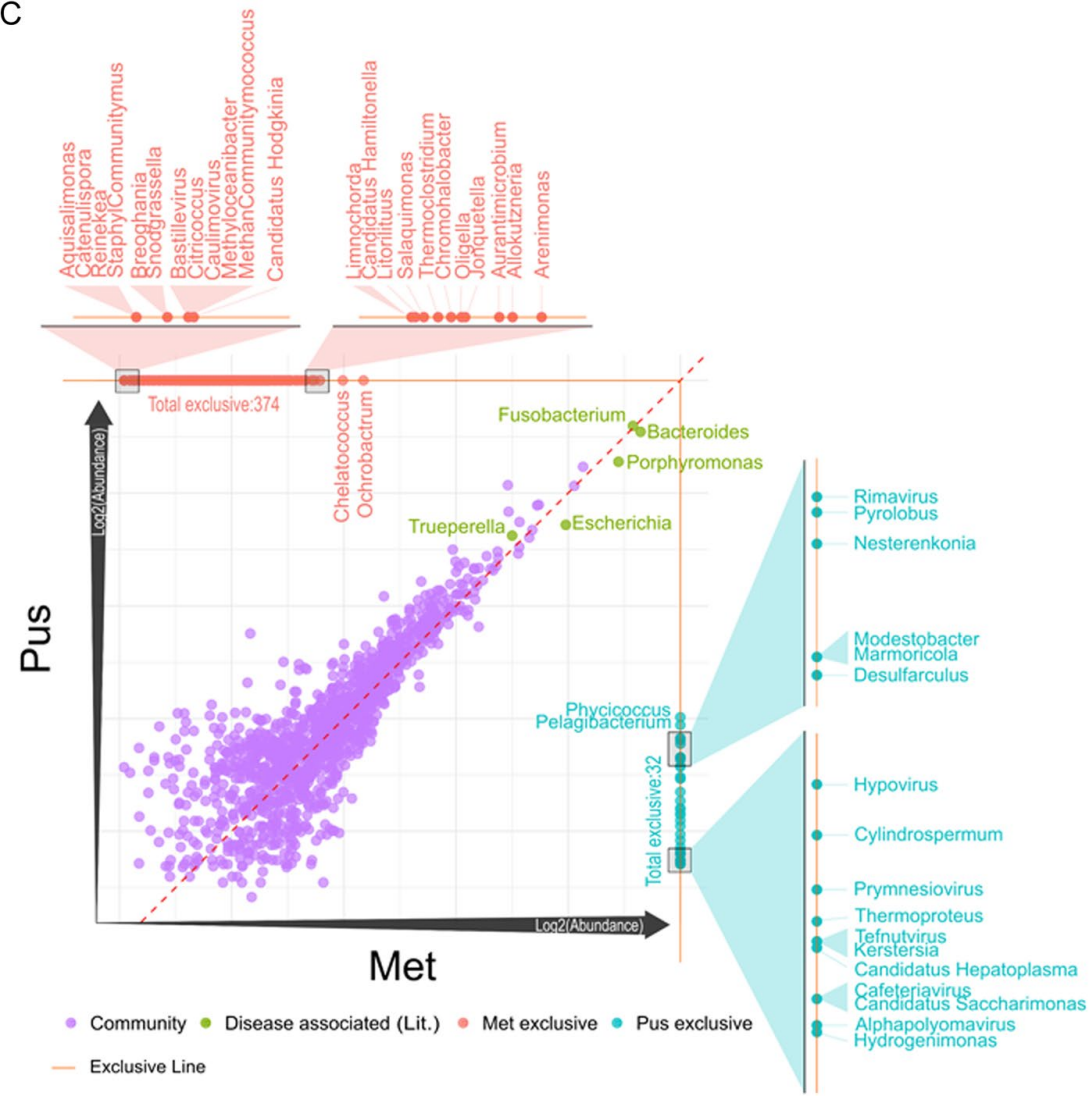
Comparison plots for the read counts at the genus level across three distinct treatment groups: control (CT), metritis (Met), and pus (Pus). The dataset was then segmented for three pairwise group comparisons: **A** CT vs. Met, **B** CT vs. Pus, and **C** Met vs. Pus. To facilitate a comprehensive visual representation of these comparisons, log2 transformed scatter plots were generated using R. Several genera, namely "Bacteroides", "Porphyromonas", "Fusobacterium", "Escherichia", and "Trueperella", were designated as "Disease associated (Lit.)", based on existing literature linking these genera to disease conditions

Upon deploying scatter plot analysis (Fig. 3), we unraveled the unique microbial landscapes across various treatments, revealing striking differences in organism abundance among the control (CT), metritis (Met), and pus (Pus) groups. For the CT versus MET comparison, data points prominently clustered along the 45° reference line, indicating a group of organisms with consistent relative abundances across both treatments. However, points significantly deviating from this line flagged organisms with pronounced abundance differences between these two groups. Notably, organisms found exclusively in either CT or MET were clearly demarcated, emphasizing their contrasting microbial profiles. Specifically, 316 organisms (genera) were uniquely associated with the metritis condition compared to the control. For instance, Shigella emerged as the most abundant organism specific to the metritis condition, whereas 66 genera were exclusive to the control group.

The CT versus PUS comparison shared some similarities with the former but also revealed distinct patterns. While a major portion of organisms again aligned along the 45° line, the exclusive organisms for this comparison—231 for CT and 139 for Pus—highlighted the subtle differences in microbial compositions between them. Intriguingly, while CT-exclusive organisms saw an uptick in this comparison, those exclusive to Pus witnessed a decline.

For the MET versus PUS plot, distinctive microbial imprints corresponding to metritis and pus were discerned. The metritis condition boasted a rich diversity with 374 exclusive organisms, contrasted with Pus, which had a limited set of 32 exclusives. This observation provides invaluable insights into their respective microbial ecosystems.

Integral to these plots were select genera, namely Bacteroides, Porphyromonas, Fusobacterium, Escherichia, and Trueperella, designated as "Disease associated (Lit.)" in recognition of their documented links to disease conditions in the existing literature. Their positions in the scatter plots underscore their ubiquity and high abundance in both conditions across all pairwise comparisons. This allows users to hover over individual data points, accessing detailed information about each organism, thereby offering an enriched visualization experience beyond the traditional static displays.

The Venn diagram (Additional file 1: Fig. 1) crafted to elucidate shared and unique microbial constituents among the CT, MET, and PUS groups further nuanced our understanding of the microbial landscape. Central to this visualization was a significant intersection of 1,113 organisms shared across all three treatments, reflecting a core microbial community integral to all conditions. In contrast with the earlier scatter plots that underscored the abundance and specificity of organisms across pairwise comparisons, the Venn diagram shed light on intersections and exclusivities. For instance, while the scatter plots demarcated organisms exclusive to CT or Met conditions, the Venn diagram quantified this exclusivity: CT boasted 48 unique organisms, whereas MET had a more expansive unique set with 191 organisms. PUS, in comparison, had a more constrained unique microbial signature with only 14 exclusive organisms.

Diving deeper into shared microbial constituents, 183 organisms were found at the intersection of CT and MET, whereas the CT and PUS overlap housed 18 organisms (Additional file 1: Fig. 1). The MET and PUS comparison again revealed a considerable shared set, tallying 125 organisms. In terms of comprehensive microbial diversity, CT, MET, and PUS treatments comprised 1,362, 1,612, and 1,270 total organisms, respectively. This aligns with the scatter plot observations, which showed considerable organism variations across treatments, further confirmed by the unique and shared entities in the Venn diagram.

### Phyla level changes in natural log fold change in abundance

To determine if any taxa were differentially abundant between clinical groups, ANCOM-BC was used to generate natural log fold change (LFC) in abundance data. Figure 5 presents these data at the phyla level for three clinical group comparisons: MET_No_Treatment, MET_Treatment, and PUS clinical groups compared to CT. For MET_No_Treatment samples, seven phyla had increased natural LFC in abundance (Ignavibacteriae, Gemmatimonadetes, Deferribacteres, Chlorobi, Chlamydiae, Bacteroidetes, and Aquificae), and the phylum Actinobacteria has a decreased natural LFC in abundance when compared to CT. The MET_Treatment samples, when compared to CT, had the greatest number of increased (natural LFC > 0) (n = 14) and decreased (natural LFC < 0) (n = 12) phyla. Of these phyla, three were increased by one natural LFC or greater (Thermotogae, Planctomycetes, and Chlorobi) and three were decreased by one natural LFC or greater (Evosea, Euglenozoa, and Apicomplexa) when compared to CT. Lastly, PUS samples had four phyla with increased natural LFC in abundance (Gemmatimonadetes, Chlorobi, Chlamydiae, and Bacteroidetes) and the phylum Actinobacteria has a decreased natural LFC in abundance when compared to CT.

### Genus level changes in natural log fold change in abundance

Of the 12 most abundant genera, *Dietzia* and *Microbacterium* were significantly decreased in abundance in MET when compared to CT. Seven genera, namely *Bacteroides, Clostridium, Fusobacterium, Phocaeicola, Porphyromonas*, *Prevotella,* and *Streptococcus* were significantly increased in abundance in MET when compared to CT. The three genera that were unchanged in MET samples compared to CT were *Escherichia, Histophilus,* and *Trueperella.*

To compare significantly increased or decreased abundances for genera for all three clinical group comparisons, a heatmap was created (Fig. 5). Seven genera (*Liquorilactobacillus*, *Cyclobacterium*, *Owenweeksia*, *Anoxybacter*, *Flavihumibacter*, *Dyadobacter*, and *Oceanobacillus*) were increased in natural LFC abundance (minimum = 0.35 and maximum = 0.66) for MET_No_Treatment, MET_Treatment, and PUS clinical groups compared to CT. Conversely, three genera (*Pseudonocardia*, *Glutamicibacter*, and *Rathayibacter*) decreased in natural LFC abundance (minimum = − 0.54 and maximum = − 0.88) for all three clinical groups when compared to CT.

To determine whether genera often associated with metritis or uterine health by other studies were differentially abundant within our sample population, a heat map was created in Fig. 6 to analyze natural LFC in the abundance of MET_No_Treatment and MET_Treatment compared to CT for 31 selected genera. None of the selected genera were significantly changed in natural LFC abundance for Pus when compared to CT.

For MET_No_Treatment, 21 genera (e.g. *Trueperella*, *Prevotella*, *Porphyromonas*, *Fusobacterium*, *Escherichia*, and *Bacteroides*) were not significantly different when compared to CT. Four genera (*Streptobacillus*, *Filifactor*, *Citrobacter*, and *Chlamydia*) were significantly increased in natural LFC of abundance in MET_No_Treatment samples when compared to CT. Six genera (*Streptomyces*, *Micrococcus*, *Corynebacterium*, *Brucella*, *Brevibacterium*, and *Bifidobacterium*) were significantly decreased in natural LFC of abundance in MET_No_Treatment samples when compared to CT.

For MET_Treatment, four genera (e.g. *Porphyromonas*, *Fusobacterium*, *Enterococcus*, and *Chlamydia*) were not significantly different when compared to CT. Ten genera (e.g. *Streptobacillus*, *Salmonella*, *Filifactor*, *Cryptococcus*, *Citrobacter*, *Arcanobacterium*, and *Anaerococcus*) were significantly increased in natural LFC of abundance in MET_Treatment samples when compared to CT. Seventeen genera (e.g. *Trueperella, Streptomyces*, *Staphylococcus*, *Prevotella, Micrococcus*, *Klebsiella*, *Escherichia, Corynebacterium*, *Brucella*, *Brevibacterium*, *Bifidobacterium, Bacteroides,* and *Bacillus*) were significantly decreased in natural LFC of abundance in MET_Treatment samples when compared to CT. Of these 17 genera, only *Micrococcus* was decreased by one natural LFC or greater in MET_Treatment when compared to CT. Ignoring stratification by antimicrobial treatment, three genera (*Streptobacillus, Filifactor,* and *Citrobacter*) were increased and six genera (*Streptomyces, Micrococcus, Corynebacterium, Brucella*, *Brevibacterium*, and *Bifidobacterium*) were decreased in natural LFC of abundance in MET samples compared to CT.

## Discussion

### Comparison to previous shotgun metagenomics studies

This study represents the currently largest cross-sectional metagenomic characterizations of the uterus of cows with and without metritis, with a total of 95 animals from 24 commercial dairy farms. Furthermore, the data generated represents the deepest current sequencing coverage for the uterine microbiome of dairy cows, using high sequencing depth for individual samples. Together, the findings from our study represent a broad population of dairy farms in California, with a genera-level characterization and comparison of the microbiota between cows with and without metritis. Most research in bovine metritis and uterine microbiota has relied on amplicon 16S rRNA sequencing [21, 22]. As of early 2023, only one study, conducted by Bicalho et al. (2017), analyzed the bacterial microbiota present in the bovine uterus using shotgun metagenomics [6]. Their study performed shotgun metagenomics sequencing on uterine swabs samples collected from 20 cows (nine healthy and eleven metritis cows) located on a single dairy farm. Their study resulted in 6.3 million quality-filtered reads that were then passed through MG-RAST [23] annotation and relied on the alignment of 16S rDNA genes to classify bacteria, producing nearly 3 million bacterial reads. Of these nearly 3 million reads, 25,334 reads matched 16S sequences at a 97% similarity level. In contrast, our study of 95 samples resulted in 1.39 trillion raw reads and 1.34 trillion quality-filtered reads that were then classified through Kraken2 [24]. Genus-level abundance data was then created using Bracken [25] resulting in 24,616,858 Kmers assigned to a genus for identification accuracy. The high sequencing depth of the current study comparatively resulted in nearly a thousand-fold increase in the number of reads matching a bacterial taxon when compared to the Bicalho et al. study. Additionally, the transition from bacterial identification by alignment of reads towards 16S sequences to the use of exact alignment of *k*-mers allowed more accurate taxonomic identification of bacterial sequence data [26]. Furthermore, MG-RAST advises against classification past the genus level, while both Kraken2 and Bracken provided accurate species-level identification [27]. The majority of the analyses presented here were restricted to genus enabling comparison to other studies on the uterine microbiome characterization during metritis, with analysis at the species level pending.

### α and β diversity of the intrauterine microbiome

Analysis of α-diversity and β-diversity was conducted to assess variation in the type or abundance of microbes between metritis clinical groups. Current research suggests that cows with metritis or in the process of developing metritis have a dysbiosis of the uterine microbiota characterized by homogenization of taxa and a decrease in bacterial richness [22, 28]. Figure 1A displays the Chao1 index values for the three clinical groups and the only significant difference was between CT and MET samples, with MET samples having higher mean Chao1 index values. Because Chao1 estimates of microbial richness that is heavily influenced by rare taxa, the higher Chao 1 index for cows with metritis could represent the invasion of the diseased uterus by less commonly observed microbes due to a disruption in the resistance to colonization that otherwise would be observed in a healthy uterus [29]. Conversely, Fig. 1B and C display Simpson and Shannon index values, two measures of diversity that account for not only the number of species present but also the relative abundance of each species. Lower Simpson and Shannon indices are observed for MET compared to CT, aligning with that observed in previous studies [15]. As both Simpson and Shannon α-diversity indices account for microbial richness and abundance, these indices provide a more representative estimation of the microbial community presence within samples. Notably, the lack of a significant difference for the α-diversity indices between MET and PUS samples supports the hypothesis of a low microbial abundance in the uterus of cows with abnormal vaginal discharge as used for case definition for MET and PUS here, with a wide range of opportunistic microbes. For MET, a contrasting high richness and low diversity was observed, which is the characteristic of dysbiosis.

To analyze β-diversity between clinical groups, NMDS ordinations were generated for CT, MET, and PUS clinical groups (Fig. 2A), and these same clinical groups with an additional group, MET_Treatment, generated for samples taken from MET cows that had received antimicrobial treatment due to metritis (Fig. 2B). Although the colored ellipses representing the 95% confidence intervals for the various clinical groups analyzed in Fig. 2 overlap, both ordinations in Fig. 2A and B were found to be significantly different by PERMANOVA and ANOSIM. While NMDS provides a visual approach to access β-diversity, PERMANOVA, and ANOSIM statistically test for significant differences between groups and is a mainstay of microbiome studies [30]. This discrepancy between ordination and statistical analysis of the β-diversity between cows with metritis and without metritis has also been observed by other studies [15, 17]. Interestingly, in Fig. 2B the 95% confidence intervals for CT and MET_Treatment is only slightly overlapping, with a clear separation of the two clinical groups in the 3D NMDS in Additional file 1: Fig. 3B. This suggests that the combined presence of metritis and antimicrobial treatment resulted in a significant effect in distinguishing the uterine microbiome when compared to CT. This is in agreement with the findings of Jeon et al. 2021 in which ceftiofur treatment of dairy cows with metritis leads to a decreased relative abundance of *Fusobacterium*. Conversely, Jeon et al. (2018) found *Porphyromonas* significantly increased after ceftiofur treatment [31, 32].

### Differential abundance of bacterial genera previously associated with metritis

Previous culture-independent studies, such as PCR-type methods and 16S rRNA sequencing, have observed a higher relative abundance of *Bacteroides*, *Porphyromonas*, and *Fusobacterium* in the uterine microbiota of cows with metritis, and a decrease in the relative abundance of these same genera in healthy cows [9, 28]. In addition to these three genera, species in the genera *Escherichia* and *Trueperella* continue to be pathogens of interest in the etiology of bovine metritis [21]. Figure 3 confirmed *Bacteroides*, *Porphyromonas*, *Fusobacterium, Escherichia,* and *Trueperella* as genera present more abundantly in MET when compared to CT; however, the difference in mean relative abundances for these genera between CT, and MET was not significant (adjusted *p* > 0.05) (Table 1). To further investigate if these five genera, and 26 additional genera of interest, were differentially abundant between clinical groups a heatmap of natural LFC in abundance was created using data from ANCOM-BC analysis (Fig. 6). Notably, none of these five genera previously identified as present at a higher prevalence were found to be significantly different in abundance when comparing MET_No_Treatment to CT (Fig. 6, Additional file 2: Table 3). A significant decrease in abundance of *Bacteroides*, *Escherichia,* and *Trueperella* in MET_Treatment compared to CT cows was observed, suggesting antimicrobial treatment with antibiotics (the most commonly used in the farms sampled was ceftiofur), was associated with a lower bacterial load of these genera.

**Table 1:** Natural log fold change of abundance data collected from ANCOM-BC for MET vs. CT for top 12 most abundant genera

Genera	Natural log fold change of MET vs. CT^a^	MET (Combined Treatment) mean percent relative abundance^b^
Bacteroides	0.20	1.03
Clostridium	0.09	0.61
Dietzia	− 0.19	0.61
Escherichia	–	0.63
Fusobacterium	0.20	1.03
Histophilus	–	0.63
Microbacterium	− 0.16	0.67
Phocaeicola	0.16	0.64
Porphyromonas	0.19	0.89
Prevotella	0.17	0.79
Streptococcus	0.22	0.70
Trueperella	–	0.56

^a^Natural log fold change of abundance for MET (regardless of antimicrobial treatment) vs. CT for top 12 most abundant genera

^b^Mean of percent relative abundance for top 12 genera for all MET samples, regardless of antimicrobial treatment (n = 33)

“–” Indicates genera with non-significant natural log fold change values for MET vs CT (p adjusted > 0.05)

**Fig. 4:**
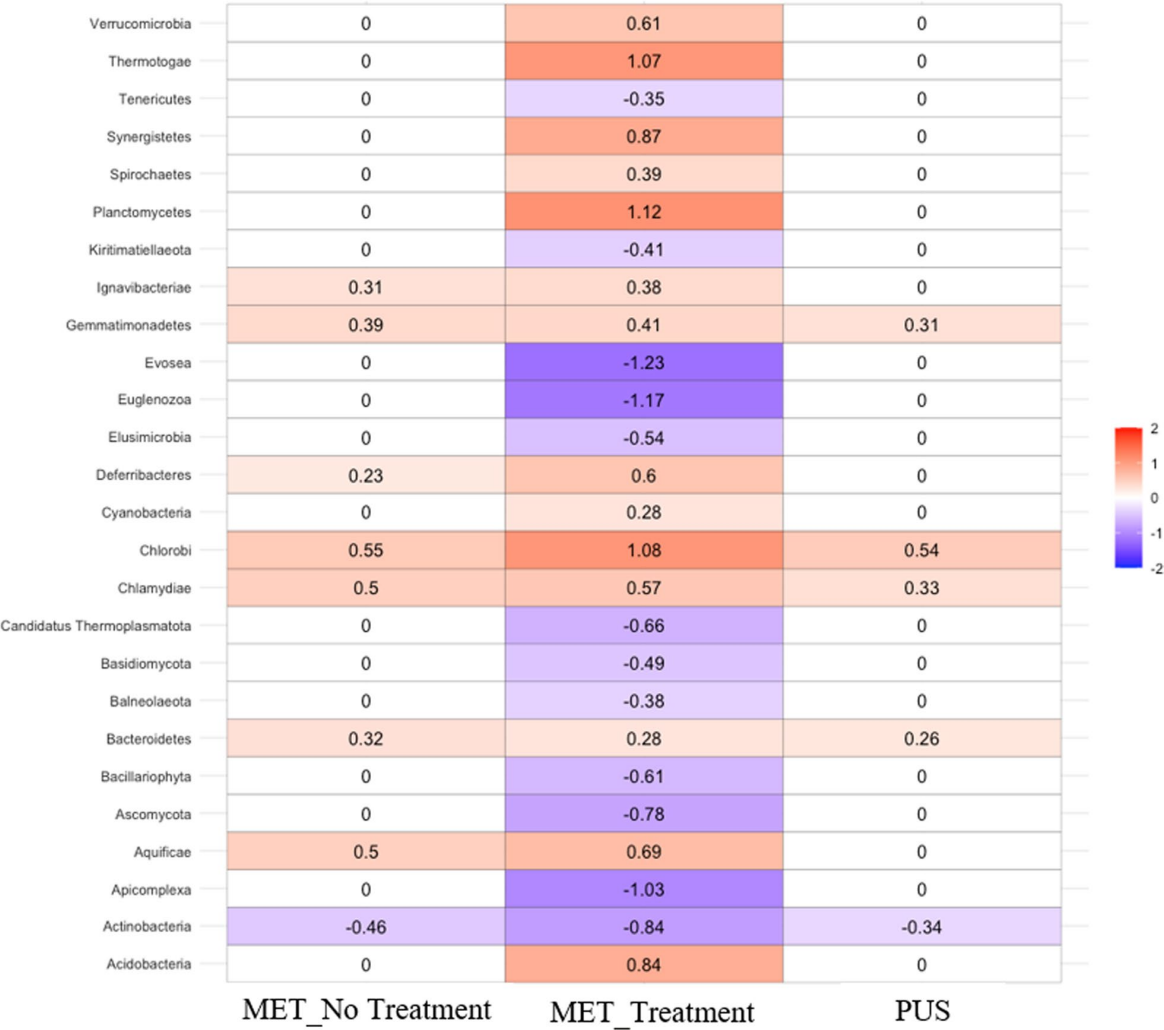
Heatmap of natural log fold changes in abundance of the 26 phyla with an adjusted *p* < 0.05 for MET_No_Treatment, MET_Treatment, or PUS when compared to control. Red indicates increased abundance in the comparison group versus control; blue indicates decreased abundance in the comparison group versus control. Zero values in white cells indicate non-significant log fold change of abundance (adjusted *p* > 0.05)

The lower abundance of *Trueperella* is further supported by the lower natural LFC in the abundance of the phylum actinobacteria (i.e., Actinomycetota), of which *Trueperella* is a member, for all clinical group comparisons versus CT (Fig. 4). This decreased abundance of *Trueperella* in MET_Treatment compared to CT cows is unsurprising as previous studies demonstrated low minimum inhibitory concentration values and resistance to ceftiofur in *E. coli* and *T. pyogenes* isolates collected from uterine swabs [20, 33]. A recent 16S rRNA-based study analyzing the impact of ceftiofur treatment on the uterine microbiome of cows with metritis revealed a significant decrease in the relative abundance of *Fusobacterium*, in contrast to our results [31]. The same study also observed that ceftiofur treatment had no significant effect on the bacterial load of *Porphyromonas* and *Bacteroides* within the uterine microbiota of cows with metritis. Interestingly, observations from Jeon et al. 2021 align with our result for *Porphyromonas*, but contrast our observed decrease in natural LFC in abundance of *Bacteroides* in MET_Treatment compared to CT cows*.* Our data are from a cross-sectional study design and limit findings to potential causal associations of individual microbes and metritis, and therefore further studies would need to be conducted to evaluate the potential causation impacts of these pathogens in the uterus following treatment or not with ceftiofur.

When comparing natural LFC of abundance for MET (regardless of antimicrobial treatment) to CT, *Bacteroides*, *Fusobacterium*, and *Porphyromonas* were increased by 0.2, 0.2, and 0.19 natural LFC, respectively (Table 1). *Escherichia* and *Trueperella* were not significantly different in natural LFC of abundance (adjusted *p* > 0.05). The observed higher natural LFC in the abundance of *Bacteroides*, *Porphyromonas*, and *Fusobacterium* in cows with metritis agrees with previous 16S rRNA and shotgun metagenomics-based studies that discovered similar findings [6, 28]. The lack of significance for *Escherichia* may be explained by the role different strains of *E. coli* play in the pathogenesis of metritis and by the decreasing likelihood of *Escherichia coli* identification following parturition, however, this should be elucidated through a study focusing on WGS of *E. coli* in cows with metritis [34, 35]. Our observed lack of significance for *Trueperella* contrasts previous 16S rRNA -based studies that reported the genus as more abundant in cows with metritis than cows without metritis [13, 15].

Two additional genera, *Micrococcus* and *Filifactor* are also of interest due to their large change in abundance, as contrasted by other genera, when comparing MET to CT (Fig. 6, Additional file 2: Table 3). Specifically, *Micrococcus* was decreased in natural LFC in abundance (-0.66 and -1.33) and *Filifactor* was increased in natural LFC in abundance (0.53 and 0.94) when comparing MET_No_Treatment and MET_Treatment to CT, respectively. Unlike the five genera previously discussed, both *Micrococcus* and *Filifactor* have not commonly been associated with bovine metritis. A culture-based study of the uterine microbiome of cows with and without metritis at the time of insemination found *Micrococcus* to be the 5^th^ most isolated genus of bacteria with 7.8% of cows being culture-positive [36]. *Micrococcus luteus* was also the 2^nd^ most commonly isolated species in the study explaining why the authors found low species diversity within this genus. Another culture-based study of the uterine microbiome also isolated *Micrococcus luteus* from cows with and without metritis (n = 6 out of 279) [37]. The lower natural LFC in the abundance of *Micrococcus* in MET cows compared to CT may be due to a lower overall bacterial diversity of the cow's uterus during metritis versus without metritis, as already described above. Bacteria in the genus *Filifactor* have been either associated with or found in high abundance in cows with metritis [9, 10]. In particular, Jeon et al. 2015 observed a significant association between *Bacteroides* and *Filifactor* in cows with metritis. Little is known about the role *Filifactor* in the development of metritis, however the species *Filifactor alocis* has been cited as an emerging pathogen in the development of human periodontal disease, a polymicrobial disease affecting the tissues around the teeth [38, 39]. As bacteria in the genus *Filifactor* have been previously associated with metritis and have been implicated in the development of another disease with a multifactorial etiology, further research into the role of *Filifactor* within the uterine microbiome may prove insightful.

### Differential abundance of bacterial genera significant for all clinical group comparisons

**Fig. 5:**
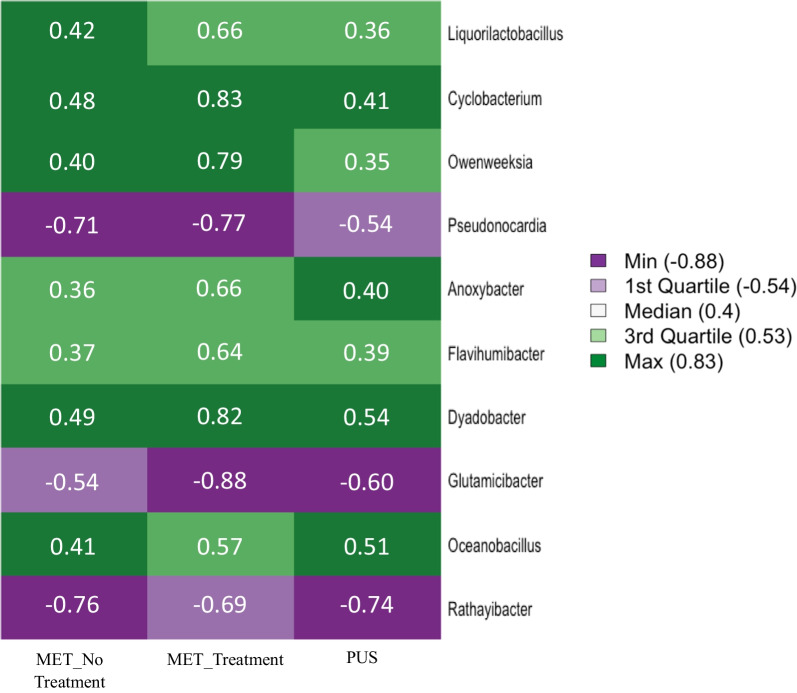
ANCOM-BC heatmap of natural log fold changes in abundance of 10 genera for which pairwise comparisons between MET_No_Treatment, MET_Treatment, and PUS when compared to CT had an adjusted *p* < 0.05. Colors correspond to 25% increments of natural log fold changes in abundance. Purple indicates decreased abundance in the comparison group versus the control. Green indicates increased abundance in the comparison group versus the control. A star indicates non-significant (adjusted *p* > 0.05) for the clinical group comparison for that genus

Ten genera were found to be significantly (*p*-adjusted < 0.05) increased or decreased in natural LFC of abundance for all three clinical group pairwise comparisons (MET_No_Treatment, MET_Treatment, and PUS when compared to CT) and are presented in Fig. 5. Seven genera (*Liquorilactobacillus*, *Cyclobacterium*, *Owenweeksia*, *Anoxybacter*, *Flavihumibacter*, *Dyadobacter*, and *Oceanobacillus)* were found to be increased in natural LFC of abundance for all three clinical group comparisons suggesting an association with uterine disease.

Members of the genus *Liquorilactobacillus* are lactic acid bacteria most often isolated from fermented plant materials (e.g. ciders, molasses, cocoa beans, and olives) [40]. Many strains of *Liquorilactobacillus* are capable of producing the exopolysaccharide dextran from sucrose [41]. Interestingly, *Liquorilactobacillus satsumensis* isolated from water kefir (also known as tibicos), a fermented beverage produced from incubating water kefir grains in water with added sugar and fruits, produced an exopolysaccharide, that when hydrolyzed, was observed to promote the growth of *Bacteroides* within an ex vivo model of the large bowel [42]. This synergy between *Liquorilactobacillus* and *Bacteroides,* a genus frequently associated with metritis [9, 28], may explain why *Liquorilactobacillus* was increased in natural LFC of abundance within cows presenting with uterine disease.

The first reported isolation of the genus *Oceanobacillus* was from deep-sea sediment at a depth of 1050 m in 2001 [43]. Since then the genus has been detected in various environmental samples, including Korean Kimchi, human gut, and pork at slaughter [44–46]. In general, *Oceanobacillus* are Gram-positive, obligate aerobes or facultative anaerobes, and moderately halophilic rods. One isolate taken from pork samples was positive for the antimicrobial resistance genes *bla*_TEM_ and *bla*_CTX-M_ that confer resistance to β-lactam antibiotics [46]. *Oceanobacillus* was detected previously in uterine samples of cows with metritis and cows without metritis but appears to be a rarely identified member of the uterine microbiome [16].

The other five genera found to be increased in natural LFC of abundance for all three clinical group comparisons have not previously been associated with the bovine uterine microbiome, suggesting they may have been contaminants in our samples. More research is needed to reveal why bacteria in the genera *Cyclobacterium* [47], *Owenweeksia* [48], *Anoxybacter* [49], *Flavihumibacter* [50], and *Dyadobacter* [51] were detected in our uterine swab samples.

The genera *Pseudonocardia*, *Glutamicibacter*, and *Rathayibacter* decreased in natural LFC abundance for all three clinical groups when compared to CT (Fig. 5). *Pseudonocardia* are aerobic, Gram-positive, non-motile bacteria that may form hyphae and have primarily been isolated from soil and other environmental samples [52]. The genus has been identified in 16S rRNA-based studies dealing with the microbiome of reproduction, including from vaginal and rectal swabs taken from human mothers and amniotic fluid samples from healthy human pregnancies [53, 54]. *Pseudonocardia* has also been cultured from cattle manure and was detected in low abundance (< 0.01% prevalence) in a 16S rRNA-based study of bovine uterine swabs [9, 55]. Given its low abundance within bovine uterine swabs and isolation from cattle manure, it is possible that *Pseudonocardia* was in the uterine microbiome, but could also have been an environmental contaminant.

*Glutamicibacter* are aerobic, gram-positive, rod-shaped bacteria often found in soil and often used in bioremediation [56, 57] and *Rathayibacter* are Gram-positive, aerobic, non-motile, irregularly shaped bacteria first isolated on annual grasses [58]. As primarily environmental bacteria, the role of these two genera in the bovine uterine microbiome remains unknown.

### Conclusion

Our findings support that uterine samples from cows without metritis (CT) had increased α-diversity but decreased β-diversity when compared to metritis or PUS cows, characteristic of dysbiosis. The microbiomes of CT and MET clinical groups were different at the genus level and differentiated based on antimicrobial treatment history. ANCOM-BC detected no significant difference in the abundance of *Bacteroides*, *Porphyromonas*, *Fusobacterium, Escherichia,* or *Trueperella* when comparing MET_No_Treatment to CT. However, *Bacteroides*, *Escherichia,* and *Trueperella* were decreased in abundance in MET_Treatment compared to CT. When comparing MET to CT (regardless of treatment) *Bacteroides*, *Porphyromonas*, and *Fusobacterium* were higher in natural LFC of abundance, while *Escherichia* and *Trueperella* were not significantly different in natural LFC of abundance, calling into question the previous hypothesis for the role of these two genera with bovine metritis. *Liquorilactobacillus* and *Oceanobacillus*, two genera either infrequently or not previously associated with bovine uterine disease, were found to be more prevalent in cows with metritis and have biologically plausible explanations for this observation. In summary, our findings highlight that MET cows have an increased abundance of *Bacteroides*, *Porphyromonas*, and *Fusobacterium* when compared to CT and PUS, and support the need for further studies to better understand their potential causal role in metritis pathogenesis.

## Material and methods

### Sample collection

Samples used for metagenomic analyses were collected as part of a study analyzing AMR to common antimicrobials used for the treatment of bovine metritis for which the University of California Institutional Animal Care and Use Committee (IACUC; #20,620) approved all experimental procedures conducted with animals and the UC Davis Institutional Review Board (IRB) Administration granted an exemption (IRB ID 1307716–1) [20].

Our population-based epidemiological used a cross-sectional study designed to collect uterine swabs from post-partum cows between 3 and 21 days in milk (DIM). A convenience sample from 25 commercial dairy farms were selected as the study population to represent the target population of dairy farms in California (Additional file 2: Table 4). For this purpose, farms sampled were located in Northern California region, the Sacramento region, and the Central Valley, which represent over 95% of milking dairy cattle in California [59]. California has over 5.1 million lactating dairy cows and is the State with the largest population of dairy cattle in the U.S. [59].

Cows sampled were classified into three clinical groups based on vaginal discharge (VD) characteristics: [60] normal discharge (**CT)**: clear lochia, clear mucus, or no vaginal discharge, metritis discharge (**MET)**: watery, reddish or brownish, and fetid, and purulent discharge (**PUS)**: non-fetid purulent or mucopurulent vaginal discharge. Samples were collected based on the diagnosis conducted for each animal immediately prior to the sample, which is described in detail in a prior publication for this study population [20]. Briefly, researchers (R.V.P. and A.G.) collected vaginal discharge from cows using a Metricheck™ device (Simcrotech, Hamilton, New Zealand) cleaned with 2% chlorhexidine gluconate solution between cows. The VD was evaluated by sight and smell, and cows were assigned to the corresponding clinical presentation group. A sub-group was also evaluated in the study for the MET group, with MET_Treatment representing MET sampled cows that had received individual antimicrobial treatment in the last 14 days prior to sampling, and MET_No_Treatment for MET sampled cows that did not receive individual antimicrobial treatment in the last 14 days prior to sampling; further description of sampling approach and treatments can be found in previously published manuscript for this study population [20]. As a population-level cross-sectional epidemiological study, a limitation of our study is that detailed information on the dosage, number of treatments, and route of administration for treatments received by cows was not available, and therefore evaluated as a combined treatment effect, and the unbalanced enrollment of cows per treatment group at the farm level (Additional file 2: Table 4). As a population-based cross-sectional study, the goal was to sample multiple farms to provide a study population that represented the diversity of the target population (CA dairy farms), and a limitation of this type of sampling approach is that no associations from herd-level farm management practices can be drawn.

Researchers cleaned the vulva using dry paper towels and 70% isopropyl alcohol prior to uterine swab collection. A 30-inch double-guarded sterile culture swab (McCullough; Jorgensen Labs Inc., Loveland, CO, USA) was gently passed through the vulva and cervix until reaching the uterine body. The swab was exposed and rolled against the uterine wall, retracted within the double sheath, removed from the cow, and placed immediately in sterile cryogenic tubes (Thermo Scientific™ Nalgene™, Rochester, NY). These tubes were transported on ice until storage in the laboratory at -80℃ for future DNA extraction.

### Sample selection

To select 95 out of the 307 swabs collected, certain criteria were created to prioritize swabs that would undergo DNA extraction and sequencing. We selected swabs collected from cows less than or equal to 14 DIM at sampling (n = 224). When possible, one swab from a MET cow matching the DIM criteria was randomly selected from each of the 25 farms enrolled in the study. After the selection of a MET cow, swabs from CT and PUS cows from the same farm were randomly selected for each of the 25 farms using a random number generator (Excel; Microsoft Corp., Redmond, WA, USA). Due to DNA quality issues, swabs chosen for sequencing were biased towards those acquired towards the end of the collection period as preliminary DNA extractions revealed more recent swabs resulted in much higher quality gDNA. After final selection (n = 95), samples were chosen from cows belonging to CT (n = 31), MET (n = 34), and PUS (n = 30) clinical groups and belonged to 24 of the 25 farms sampled in the initial study.

### DNA extraction

DNA was extracted using the QIAamp DNA minikit (Qiagen, Valencia, CA) according to the manufacturer’s instructions for buccal swabs. The addition of the following steps to increase the recovery of high-quality DNA was based on methodology from previous publications [61]. Frozen swabs were placed in sterile 2 mL microcentrifuge tubes with 400 µL of buffer AL and left to thaw at room temperature for 30 min. Once thawed, the swab was removed with sterile forceps and the microcentrifuge tube was centrifuged at 13,200 × g for 10 min before the supernatant was discarded and the pellet re-suspended in 245 µL of buffer AL. 5 µL of lysozyme (50 mg/mL; ThermoFisher Scientific, Waltham, MA) and 150 µL of mutanolysin diluted to 1000 Units/mL from *Streptomyces globisporus* ATCC 21553 (Sigma-Aldrich, Saint Louis, MO,) were added to each sample before incubating at 37℃ for one hour. DNA extraction was performed as specified by QIAamp DNA minikit protocol. To ensure adequate purity for DNA sequencing, eluted DNA was purified according to the Zymo Genomic DNA Clean & Concentrator kit (Zymo Research, Irvine, CA). After subsequent DNA extraction and purification, DNA quantification was conducted for all samples using a NanoDrop One^C^ (Thermo Fisher Scientific, Wilmington, DE).

### Library prep and metagenomic sequencing

Illumina DNA libraries were prepared using the seqWell plexWell LP384 Library Preparation kit (seqWell, Beverly, MA) using 10 ng of genomic DNA. The prepared libraries were amplified with 8 PCR cycles, analyzed using Bioanalyzer 2100 (Agilent, Santa Clara, CA), quantified with Qubit (Life Technologies, ThermoFisher Scientific, USA), and combined into one pool at equimolar ratios. The library pool was quantified by qPCR with the Kapa Library-Quant kit (Kapa Biosystems/Roche, Basel, Switzerland) and sequenced on an Illumina NovaSeq system (Illumina, San Diego, CA) with paired-end 150-bp reads.

### Bioinformatics

Raw sequence data were trimmed (Nextera adapters and low-quality sequence) using Trimmomatic (version 0.39; command: trimmomatic PE [input] [output] ILLUMINACLIP:[adapters]:2:40:15 LEADING:2 TRAILING:2 SLIDINGWINDOW:4:15 MINLEN:50) [62] and reads assessed for quality at each step using FastQC (version 0.11.9). Trimmed and quality filtered reads were sorted into bovine and non-bovine reads using the sorting function “classified-out” in Kraken2 (version 2.0.8) [24], with bovine genome reference (USDA ARS-UCD1.2; RefSeq assembly accession: GCF_002263795.1). Microbial reads were identified from non-bovine reads using the classification function in Kraken2, using kraken database of microbes from RefSeq, built [May 5, 2021] using standard Kraken 2 database module reference libraries (archaea, bacteria, viral, fungi, protozoa, UniVec_Core), as previously described [63]. Microbial reads were calculated to form phyla, genus, and species level taxa abundances using Bracken (version 2.6.1; Bracken database compiled from microbial Kraken2 database used, constructed for k-mer size 35nt and read length 150nt) [25].

### Shotgun metagenomics sequence data and availability

Sequencing resulted in a total of 1,385,735,271 raw paired-end reads. After quality trimming, removal of bovine sequence, and assignment of microbial reads to genera, 95 samples resulted in a total of 24,616,858 reads prior to CSS normalization. The mean number of genus-level reads per sample was 256,425 (95% CI: 124,471–388,379). After CSS normalization, 95 samples resulted in a total of 135,190 reads. The mean number of genus-level reads per sample was 1,408 (95% CI: 1,265–1,551). Sequencing reads for all samples analyzed in this project have been deposited under NCBI Bioproject PRJNA186441.

When compared to prior uterine microbial characterization using a metataxonomic approach such as 16S rRNA gene sequencing, a metagenomic approach like shotgun metagenomic sequencing (**SMS**), 16S rRNA may reveal only part of a microbial community discovered by SMS, and therefore SMS results in a novel data that has more power to identify less abundant taxa, which can be biologically meaningful, especially when comparing disease and healthy microbial communities [63].

### Diversity analysis

Cumulative Sum Scaling (CSS) was used to normalize reads via metagenomeSeq (version 1.42) in RStudio (version 4.2) at the genus-level [64, 65]. To facilitate diversity analyses, prior to the creation of a *phyloseq* object, Taxallnomy was used to create a hierarchical taxa table [66]. *Phyloseq* objects were created using both CSS-normalized and non-normalized read counts [67]. Using non-normalized data, α diversity metrics (Shannon index and Chao1) were calculated at the genus level. After testing for normality via Shapiro–Wilk test, significant differences between clinical groups was tested using the Wilcoxon Sum Rank test for Chao1 and Simpson values and the Tukey–Kramer HSD test for Shannon values in JMP Pro 16. The α diversity metrics were graphed using the “alpha_boxplot” function of the Amplicon package from microbiome using RStudio (Fig. 1) [68]. *P* ≤ 0.05 was considered a significant difference.

β diversity was visualized at the genus-level using Bray–Curtis dissimilarity distances calculated from CSS normalized read count data using nonmetric multidimensional scaling (NMDS) using the “ordinate” function of *phyloseq* (version 1.40) in RStudio [67]. Ordination fit was assessed using stress values and when stress values ≥ 0.2 were obtained, NMDS was repeated with an increased *trymax* of up to 200 until stress values < 0.2 were obtained. Two NMDS ordinations were created, Fig. 2A analyzing β diversity between clinical groups of cows sampled (CT = 31, MET = 34, and PUS = 30) and Fig. 2B analyzing β diversity between the same clinical groups, but with samples from MET cows separated by whether cows sampled received antimicrobial treatment within fourteen days prior to intrauterine swab collection (CT = 31, MET_No_Treatment = 25, MET_Treatment = 9, and PUS = 30). Colored ellipses were added to represent the 95% confidence interval for the various clinical groups. Three-dimensional scatterplots of both NMDS ordinations (Additional file 1: Fig. 3) were created using the “beta_diversity_3d” function of *plotly_microbiome* (version 0.0.9) [69].

For both NMDS ordinations, significant differences were tested by performing permutational multivariate analysis of variance (PERMANOVA) using 999 permutations and by analysis of similarities (ANOSIM) via the “adonis2” function of the *vegan package* (version 2.6–4) within RStudio. For PERMANOVA, differences were considered significant when *p*-adjusted < 0.01. For ANOSIM, differences were considered significant when *p*-adjusted < 0.01. For PERMANOVA, post hoc pairwise comparisons between groups (Additional file 2: Table 4–1) were conducted using “pairwise.adonis” with *P* values adjusted using Benjamini–Hochberg [70]. Following PERMANOVA, the “betadisper” function was used to test for homogeneity of multivariate dispersion for NMDS of clinical groups for cows sampled and for NMDS of clinical groups, but with samples from MET cows separated by antimicrobial treatment. Pairwise ANOSIM between clinical groups of cows sampled and between clinical groups, but with samples from MET cows separated by antimicrobial treatment was also conducted with *P* values adjusted using Benjamini-Hochberg (Additional file 2: Table 2).

Taxa distribution outputs, from analysis with Kraken2/Bracken, were graphed for supplemental figures by a python script using the following packages: Pandas (version 2.0.1, dataframe functions), Seaborn (version 0.12.2, heatmap function), Matplotlib (version 3.7.1, figure alterations), and are presented as a heatmap in Additional file 1: Fig. 3.

### Venn diagram

To discern both the common and distinct organisms across the three treatment groups—metritis (Met), pus (Pus), and control (CT)—we constructed a Venn diagram. This diagram visually highlights the intersections and distinctions in organism presence among the treatment groups. Each segment of the Venn diagram corresponded to a specific treatment, with overlapping areas indicating organisms shared between treatments. In contrast, non-overlapping portions indicate organisms unique to a single treatment (Additional file 1: Fig. 1).

### Comparison plots

Read counts of genus level, derived from Bracken analysis, were normalized to ascertain relative abundance values. This was achieved by dividing the raw abundance of each organism by the total abundance within its corresponding sample. This normalized data allowed us to compute average abundances across three distinct treatment groups: control (CT), metritis (Met), and pus (Pus). The dataset was then segmented for three pairwise group comparisons: CT vs. Met, CT vs. Pus, and Met vs. Pus (Fig. 3). To facilitate a comprehensive visual representation of these comparisons, log2 transformed scatter plots were generated using R. Several genera, namely "Bacteroides", "Porphyromonas", "Fusobacterium", "Escherichia", and "Trueperella", were designated as "Disease associated (Lit.)", based on existing literature linking these genera to disease conditions.

### Differential abundance testing

Analysis of Compositions of Microbiomes with Bias Correction (ANCOM-BC) (version 2.0.2) was used to detect differences in microbial compositions between clinical groups [71]. CSS normalized read count data was input into ANCOM-BC, which was selected as it provides *P* values and confidence intervals for each taxon, controls the false discovery rate, and is relatively computationally simple to implement. ANCOM-BC utilizes the Wilcoxon rank-sum test for identifying taxa that are differentially abundant and includes multiple hypothesis corrections by the Holm-Bonferroni method. It is a useful tool for comparing relative abundance between groups due to its capacity to control the false discovery rate at nominal levels while maintaining power. An ANCOM-BC detection q value < 0.05 was considered significant (q values are the *P* values adjusted for the optimized false discovery rate).

**Fig. 6:**
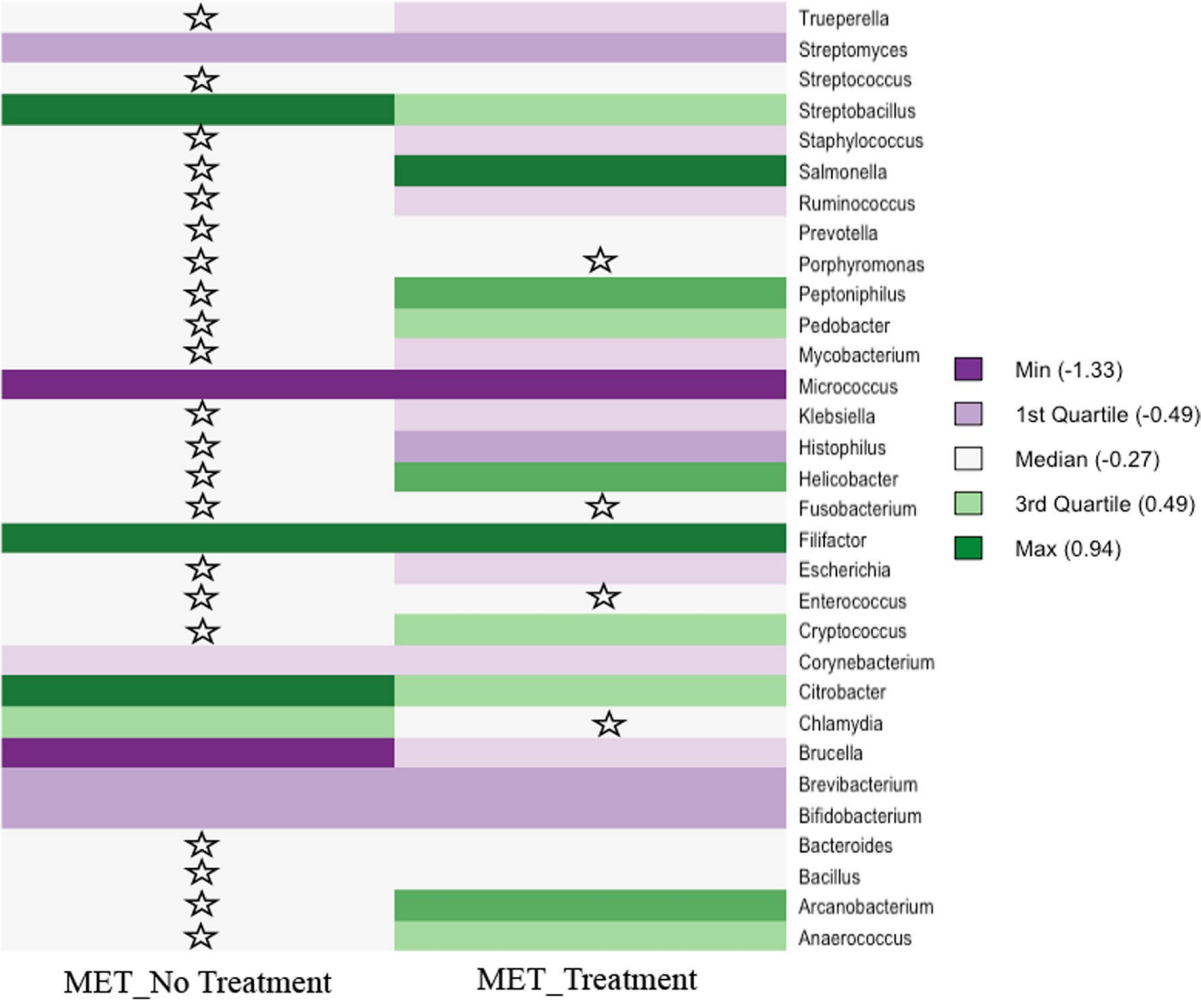
ANCOM-BC heatmap of natural log fold changes in abundance of 31 selected genera for which MET_No_Treatment and/or MET_Treatment when compared to CT had an adjusted *p* < 0.05. PUS was not included as none of the selected genera were significant for this clinical group. Colors correspond to 25% increments of natural log fold changes in abundance. Purple indicates decreased abundance in the comparison group versus the control. Green indicates increased abundance in the comparison group versus the control. A star indicates non-significant (adjusted *p* > 0.05) for the clinical group comparison for that genus

ANCOM-BC computes natural log fold changes (LFC) between groups and this data was used to create heatmaps to compare significant changes between clinical groups for various taxa either by using native ANCOM-BC code or by the “heatmap” function in RStudio. Figure 4 presents a heatmap of natural LFC of phyla abundance in MET_No_Treatment, MET_Treatment, and PUS clinical groups compared to CT. Figure 5 presents a heatmap of natural LFC of the abundance of 10 genera for which all three clinical group pairwise comparisons (MET_No_Treatment, MET_Treatment, and PUS when compared to CT) were significant (*p*-adjusted < 0.05). Figure 6 presents a heatmap of natural LFC in the abundance of 31 selected genera for MET_No Treatment and MET_Treatment clinical group pairwise comparisons when compared to CT. Data used to create Fig. 6 is presented in Additional file 2: Table 3. Table 1 displays the natural LFC in abundance, along with the mean percent relative abundance within MET samples, of the top 12 most abundant genera when MET (regardless of treatment) was compared to CT.

### Supplementary Information

**Additional file 1**: Contains additional Figures 1 to 3.

**Additional file 2**: Contains additional Tables 1 to 4.

## Data Availability

The datasets used and/or analyzed during the current study are available from the corresponding author upon reasonable request.
