# Supplementary material for: Unveiling the microbiome during post-partum uterine infection: a deep shotgun sequencing approach to characterize the dairy cow uterine microbiome
Source: Anim Microbiome. 2023 Nov 20;5:59. doi: 10.1186/s42523-023-00281-5 (PMC10662892; doi:10.1186/s42523-023-00281-5)
Supplement: Supplementary file 1 — Additional file 1: Contains additional Figures 1 to 3. [file 42523_2023_281_MOESM1_ESM.docx]

**Supplemental Figure 1**. Venn diagram displaying common and distinct organisms across the three treatment groups - metritis (Met), pus (Pus), and control (CT). Each segment of the Venn diagram corresponded to a specific treatment, with overlapping areas indicating organisms shared between treatments. In contrast, non-overlapping portions indicate organisms unique to a single treatment.


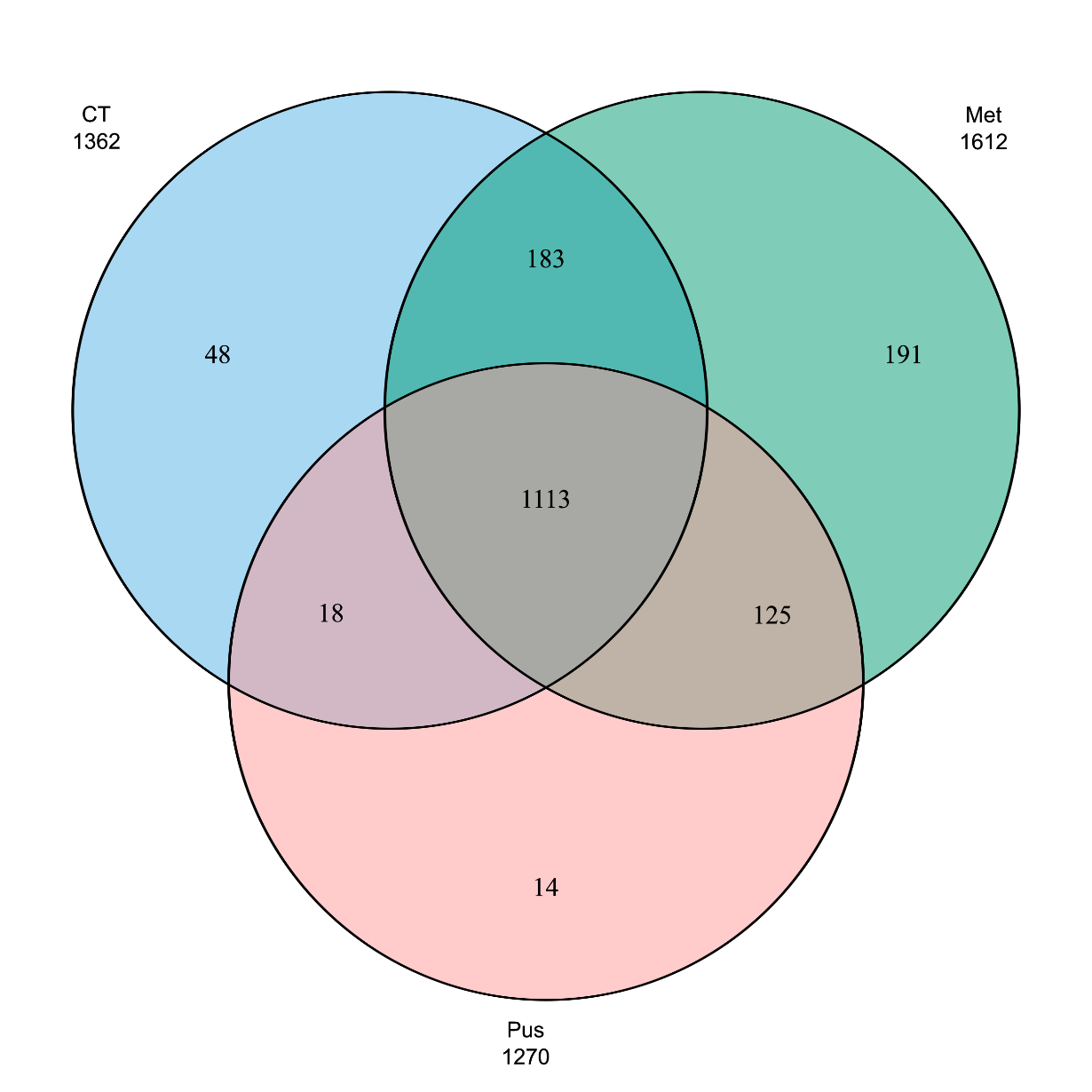


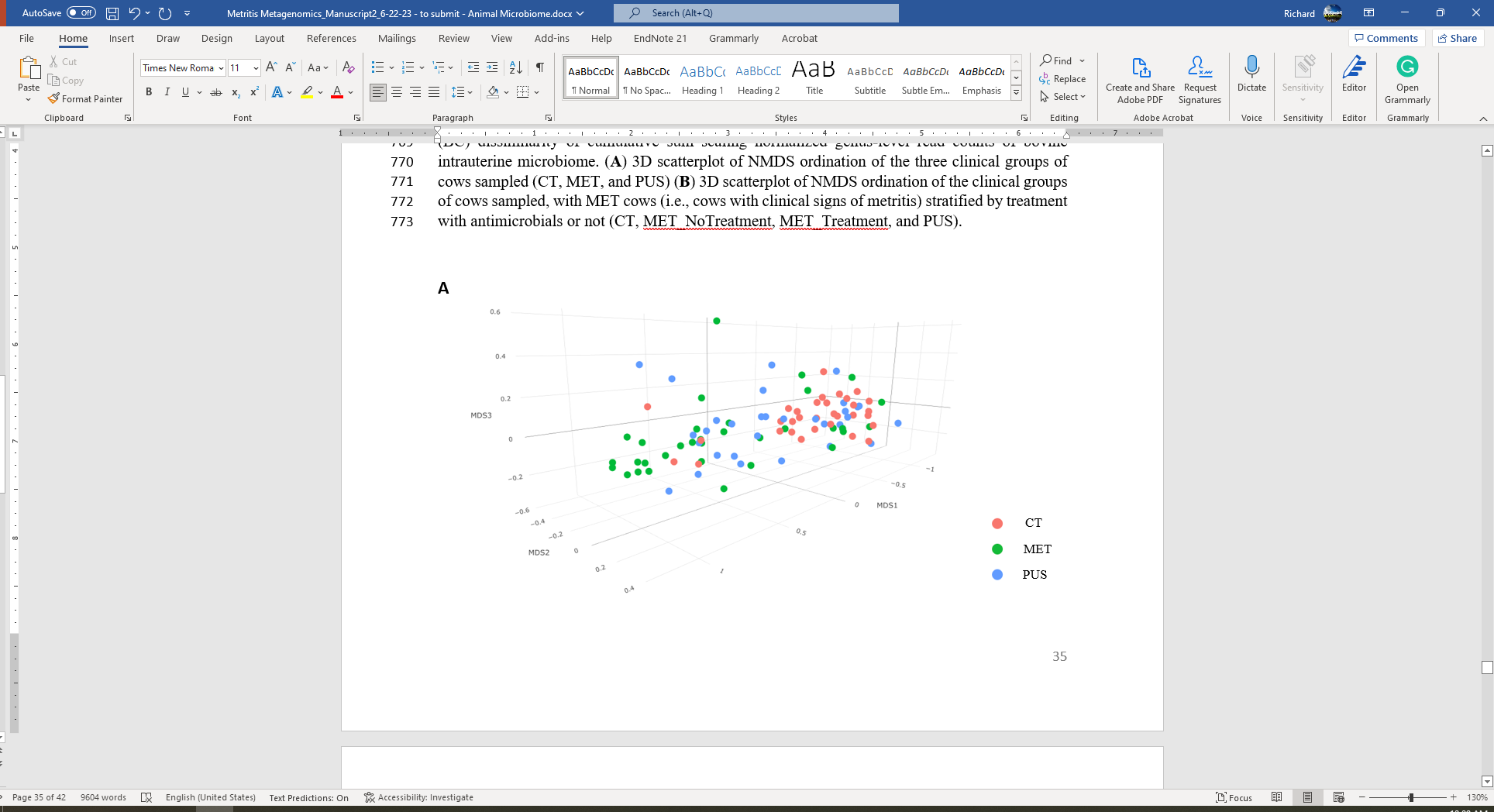
**Supplemental Figure 2**. 3D scatterplot of nonmetric multidimensional scaling (NMDS) based on Bray-Curtis (BC) dissimilarity of cumulative sum scaling normalized genus-level read counts of bovine intrauterine microbiome. (**A**) 3D scatterplot of NMDS ordination of the three clinical groups of cows sampled (CT, MET, and PUS) (**B**) 3D scatterplot of NMDS ordination of the clinical groups of cows sampled, with MET cows (i.e., cows with clinical signs of metritis) stratified by treatment with antimicrobials or not (CT, MET_No_Treatment, MET_Treatment, and PUS).


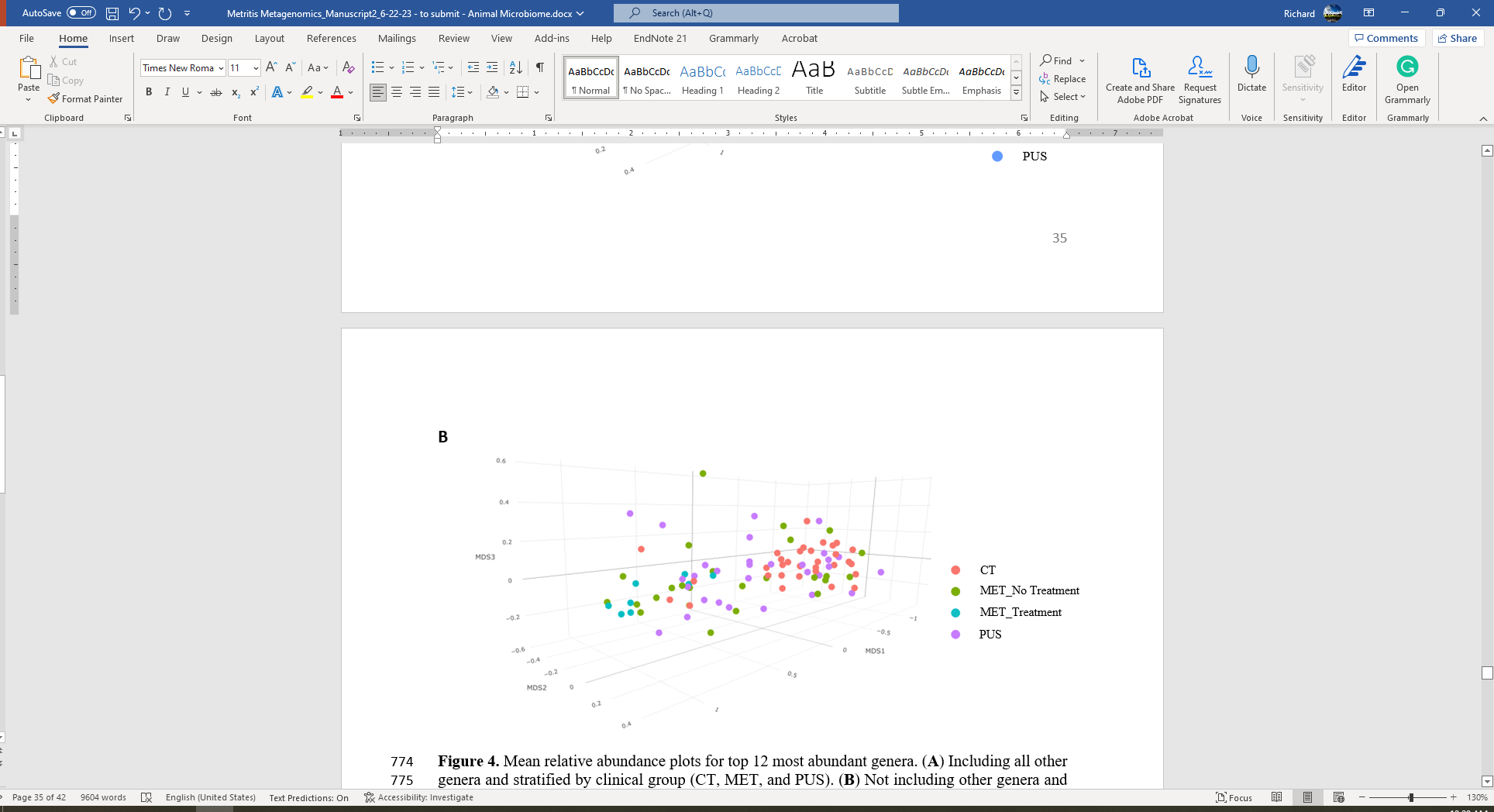


**Figure 3.** Heatmap displaying average taxa abundancies by clinical group at the genus level. Taxa counts are given as the average number of the normalized reads for each clinical group. Read count normalization is reads per million [((number of raw reads for a taxa in sample)/(total microbial identified reads for sample))*10^6^]. Taxa displayed were subset to only taxa that had at least 100 reads per million in at least one clinical group. Samples are order by taxa with the highest number of unnormalized reads across samples.
