# Supplementary material for: Unveiling the microbiome during post-partum uterine infection: a deep shotgun sequencing approach to characterize the dairy cow uterine microbiome
Source: Anim Microbiome. 2023 Nov 20;5:59. doi: 10.1186/s42523-023-00281-5 (PMC10662892; doi:10.1186/s42523-023-00281-5)
Supplement: Supplementary file 2 — Additional file 2: Contains additional Tables 1 to 4. [file 42523_2023_281_MOESM2_ESM.docx]

**Supplemental Table 1**. PERMANOVA (permutational multivariate analysis of variance) to test the significance of genus-level Bray-Curtis dissimilarity distances of different clinical group comparisons (999 permutations).

| Variable | | Degrees of Freedom | Sum of Squares | R^2^ | F | *p*-adjusted^4^ |
| --- | --- | --- | --- | --- | --- | --- |
| Clinical Groups | |  |  |  |  |  |
| CT, MET, PUS ^1^ | 2 | 0.93 | 0.08459 | 4.2972 | **0.001** |  |
| CT, MET_Treat, MET_NoTreat, PUS ^2^ | 3 | 1.391 | 0.12649 | 4.4401 | **0.001** |  |
|  | |  |  |  |  |  |
| Pairwise^3^ | |  |  |  |  |  |
| CT v. MET | 1 | 0.855 | **0.112** | 7.911 | **0.003** |  |
| CT v. PUS | 1 | 0.267 | 0.041 | 2.607 | 0.046 |  |
| MET v. PUS | 1 | 0.264 | 0.036 | 2.312 | 0.046 |  |
|  |  |  |  |  |  |  |
| CT v. MET_NoTreat | 1 | 0.491 | **0.075** | 4.485 | **0.006** |  |
| CT v. PUS | 1 | 0.267 | 0.041 | 2.607 | 0.034 |  |
| CT v. MET_Treat | 1 | 1.06 | **0.242** | 12.123 | **0.003** |  |
| MET_NoTreat v. PUS | 1 | 0.098 | 0.015 | 0.846 | 0.471 |  |
| MET_NoTreat v. MET_Treat | 1 | 0.46 | 0.12 | 4.241 | 0.011 |  |
| PUS v. MET_Treat | 1 | 0.658 | **0.155** | 6.785 | **0.003** |  |

(**1**) Comparison of clinical groups used at intrauterine swab sampling (CT, MET, and PUS). (CTL) control, healthy discharge defined as cows with either no vaginal discharge, clear mucus, or clear lochia; (MET) metritis discharge defined as a watery, red or brown colored, and fetid vaginal discharge; and (PUS) purulent discharge defined as a non-fetid purulent or mucopurulent vaginal discharge.

(**2**) Comparison of clinical groups stratified by whether MET cows received antimicrobial treatment.

(**3**) Pairwise comparisons of clinical groups

(**4**) *P* values adjusted using Benjamini-Hochberg. Significant values (*p* < 0.01) presented in bold.

**Supplemental Table 2**. ANOSIM (analysis of similarities) to test the significance of genus-level Bray-curtis dissimilarity distances of different clinical group comparisons.

| Variable | | R | Adjusted *p-*value^4^ |
| --- | --- | --- | --- |
| Clinical Groups | |  |  |
| CT, MET, PUS ^1^ | 0.1277 | **0.001** |  |
| CT, MET_Treat, MET_NoTreat, PUS^2^ | 0.1289 | **0.001** |  |
|  | |  |  |
| Pairwise^3^ | |  |  |
| CT v. MET_NoTreat |  | **0.003** |  |
| CT v. PUS |  | 0.018 |  |
| CT v. MET_Treat |  | **0.003** |  |
| MET_NoTreat v. PUS |  | 0.343 |  |
| MET_NoTreat v. MET_Treat |  | 0.648 |  |
| PUS v. MET_Treat |  | 0.168 |  |

(**1**) Comparison of clinical groups used at intrauterine swab sampling (CT, MET, PUS). (CTL) control, healthy discharge defined as cows with either no vaginal discharge, clear mucus, or clear lochia; (MET) metritis discharge defined as a watery, red or brown colored, and fetid vaginal discharge; and (PUS) purulent discharge defined as a non-fetid purulent or mucopurulent vaginal discharge.

(**2**) Comparison of clinical groups stratified by whether MET cows received antimicrobial treatment.

(**3**) Pairwise comparisons of clinical groups

(**4**) *P* values adjusted using Benjamini-Hochberg. Significant values (*p* < 0.01) are presented in bold.

**Supplemental Table 3**. Table of natural log fold changes in abundance of 31 selected genera for which MET_No_Treatment and/or MET_Treatment when compared to CT had an adjusted *p* < 0.05.

| Genera | MET_No_Treatment | Met_Treatment |
| --- | --- | --- |
| *Trueperella* | **-** | -0.43 |
| *Streptomyces* | -0.27 | -0.68 |
| *Streptococcus* | **-** | -0.15 |
| *Streptobacillus* | 0.56 | 0.51 |
| *Staphylococcus* | **-** | -0.44 |
| *Salmonella* | **-** | 0.91 |
| *Ruminococcus* | **-** | -0.40 |
| *Prevotella* | **-** | -0.20 |
| *Porphyromonas* | **-** | **-** |
| *Peptoniphilus* | **-** | 0.66 |
| *Pedobacter* | **-** | 0.35 |
| *Mycobacterium* | **-** | -0.35 |
| *Micrococcus* | -0.66 | -1.33 |
| *Klebsiella* | **-** | -0.51 |
| *Histophilus* | **-** | -0.79 |
| *Helicobacter* | **-** | 0.65 |
| *Fusobacterium* | **-** | **-** |
| *Filifactor* | 0.53 | 0.94 |
| *Escherichia* | **-** | -0.34 |
| *Enterococcus* | **-** | **-** |
| *Cryptococcus* | **-** | 0.59 |
| *Corynebacterium* | -0.23 | -0.56 |
| *Citrobacter* | 0.49 | 0.47 |
| *Chlamydia* | 0.23 | **-** |
| *Brucella* | -0.62 | -0.49 |
| *Brevibacterium* | -0.34 | -0.76 |
| *Bifidobacterium* | -0.38 | -0.74 |
| *Bacteroides* | - | -0.20 |
| *Bacillus* | - | -0.27 |
| *Arcanobacterium* | - | 0.67 |
| *Anaerococcus* | - | 0.40 |

“-” Non-significant (*p* adjusted > 0.05) for the clinical group comparison for that genus.

**Supplemental Table 4.** Count distribution of animals sampled (n=95) by clinical group (CT, MET, or PUS), and for MET sub-group by treatment (MET _No Treatment and MET_Treatment) for each of the 24 dairies where cattle were sampled.

| FARM ID | Clinical Group | | | MET Sub-Group | |
| --- | --- | --- | --- | --- | --- |
|  | **CT** | **MET** | **PUS** | **MET _No Treatment** | **MET_Treatment** |
| 1 | 2 | 0 | 0 | 0 | 0 |
| 2 | 1 | 1 | 1 | 1 | 0 |
| 3 | 2 | 1 | 1 | 1 | 0 |
| 4 | 1 | 1 | 1 | 0 | 1 |
| 5 | 1 | 0 | 0 | 0 | 0 |
| 6 | 0 | 1 | 1 | 0 | 1 |
| 7 | 0 | 2 | 1 | 2 | 0 |
| 8 | 0 | 1 | 1 | 1 | 0 |
| 9 | 1 | 2 | 0 | 2 | 0 |
| 10 | 0 | 2 | 1 | 1 | 1 |
| 11 | 1 | 0 | 1 | 0 | 0 |
| 12 | 1 | 1 | 1 | 0 | 1 |
| 13 | 3 | 3 | 2 | 3 | 0 |
| 14 | 2 | 1 | 1 | 1 | 0 |
| 15 | 1 | 2 | 1 | 2 | 0 |
| 16 | 1 | 3 | 2 | 3 | 0 |
| 17 | 1 | 1 | 2 | 0 | 1 |
| 18 | 0 | 0 | 2 | 0 | 0 |
| 19 | 1 | 3 | 1 | 1 | 2 |
| 20 | 3 | 1 | 1 | 1 | 0 |
| 21 | 3 | 1 | 3 | 1 | 0 |
| 22 | 3 | 1 | 3 | 1 | 0 |
| 23 | 1 | 5 | 0 | 3 | 2 |
| 24 | 2 | 1 | 3 | 1 | 0 |
